# Stabilization and electronic topological transition of hydrogen-rich metal Li_5_MoH_11_ under high pressures from first-principles predictions

**DOI:** 10.1038/s41598-021-83468-7

**Published:** 2021-02-18

**Authors:** Prutthipong Tsuppayakorn-aek, Wiwittawin Sukmas, Rajeev Ahuja, Wei Luo, Thiti Bovornratanaraks

**Affiliations:** 1 Extreme Conditions Physics Research Laboratory (ECPRL) and Physics of Energy Materials Research Unit, Department of Physics, Faculty of Science, Chulalongkorn University, Bangkok, 10330 Thailand; 2 Thailand Centre of Excellence in Physics, Ministry of Higher Education, Science, Research and Innovation, 328 Si Ayutthaya Road, Bangkok, 10400 Thailand; 3 Condensed Matter Theory Group, Department of Physics and Materials Science, Uppsala University, Box 530, 751 21 Uppsala, Sweden; 4 Applied Materials Physics, Department of Materials and Engineering, Royal Institute of Technology (KTH), 100 44 Stockholm, Sweden

**Keywords:** Materials science, Physics

## Abstract

Regarded as doped binary hydrides, ternary hydrides have recently become the subject of investigation since they are deemed to be metallic under pressure and possibly potentially high-temperature superconductors. Herein, the candidate structure of Li_5_MoH_11_ is predicted by exploiting the evolutionary searching. Its high-pressure phase adopts a hexagonal structure with P6_3_/mcm space group. We used first-principles calculations including the zero-point energy to investigate the structures up to 200 GPa and found that the P6_3_cm structure transforms into the P6_3_/mcm structure at 48 GPa. Phonon calculations confirm that the P6_3_/mcm structure is dynamically stable. Its stability is mainly attributed to the isostructural second-order phase transition. Our calculations reveal the electronic topological transition displaying an isostructural second-order phase transition at 160 GPa as well as the topology of its Fermi surfaces. We used the projected crystal orbital Hamilton population (pCOHP) to examine the nature of the chemical bonding and demonstrated that the results obtained from the pCOHP calculation are associated with the electronic band structure and electronic localized function.

## Introduction

The success of experimental syntheses of metal hydrides has attracted considerable attention especially amongst the high-pressure research community, a corollary to discoveries—both experimental and theoretical—of a variety of interesting physical properties of these materials^1–8^. Also, metal hydrides have long been considered as one of the best candidates for high-temperature superconductors. The superhydride Ce-H system, for instance, was successfully synthesized using the laser-heated diamond anvil cell (DAC) accompanied with synchrotron X-ray diffraction, which was theoretically confirmed by the evolutionary variable-composition simulation indicating that CeH_9_ adopts a hexagonal clathrate structure with the P6_3_/mmc symmetry and potentially produces high-temperature superconductivity with estimated $T_{c}$ of 105–117 K at 200 GPa^9^. Another metal-hydride superconductor of LaH_10_ with $T_{c}$ = 260 K, approaching room temperature, was synthesized and experimentally observed under pressure between 180–200 GPa^10^, while it was suggested by a subsequent theoretical study to adopt a sodalite-like clathrate structure with $Fm\overline{3}m$ symmetry and exhibit a decreasing trend in $T_{c}$ under an increase of pressure^11^. Additionally, LaH_11_ is expected to produce high $T_{c}$ as theoretically predicted^12^ as well as several other materials^13–19^.

More recently, ternary superhydrides (compounds each consisting of two substituted metal elements and hydrogen atoms) have been proposed to achieve metallicity and that one can expect a near room-temperature superconductivity out of them^10,11,17,18^. However, the ternary hydride Li_5_MoH_11_ does not at all exhibit high-temperature superconductivity under high pressure, only with its maximum $T_{c}$ of 6.5 K at 160 GPa belonging to the high-pressure phase^20^. Li_5_MoH_11_ being one of the family of the hydrogen-rich metals Li_5_MH_11_, where M = Mo, W, Nb, and Ta^21^, adopts a hexagonal structure with the P6_3_cm space group at ambient pressure, which is constructed via the ionic bonds of *i*.*e*. Li^+^, H^−^, and the ninefold-hydrogen-coordinated [MoH_9_]$^{3-}$. The structure was also predicted in theory to exhibit an insulator–metal transition under compression^21^. As for Li_5_MoH_11_, it was revealed that the P6_3_cm structure transforms into the *Cc* and *Pc* structures at 5 GPa and 94 GPa, respectively^21^. Not long ago an experimental study utilizing high-pressure synchrotron x-ray diffraction (XRD) cast doubt on the ambiguity in structural determination of this ternary compound. According to the observation, the *Cc* and *Pc* structures do not exist at the predicted pressures (5 GPa and 94 GPa, respectively) as well as the fact that at higher pressure the crystal structure of Li_5_MoH_11_ cannot also be determined experimentally^20^.

In this work, we aimed to identify the unknown structure of Li_5_MoH_11_ above 160 GPa, taking the crystal structure obtained from experiment as the starting structure throughout the entire calculation, by using the Universal Structure Predictor: Evolutionary Xtallography (USPEX). It is well-known that the standard spin-polarized DFT calculation, specifically within the generalized gradient approximation (GGA functional), cannot successfully describe the *d*-orbital of a transition metal^22^, e.g. Mo atom^23^, due highly to the strongly correlated orbitals. In order to fully take into account the valence states of Mo atom, we performed the GGA+U calculations with the objective of correctly determining the reliable values of $U_{\text {eff}}$ for Li_5_MoH_11_. Regarding its potential for superconductivity, the electronic structure and the nature of the chemical bonding observed by the experimental study^20^ were shown to propound a possibility of $T_{c}$. Again, the aforementioned experimental observation found that Li_5_MoH_11_ has two different superconducting phases, indicating that a phase transition at 160 GPa might be expected to be an isostructural second-order phase transition, while the possibility of which is supported by means of an electronic topological transition (ETT). The concept of ETT has successfully been used to describe the nature of the electronic structures in several materials^24–28^, particularly the metal hydride class^24^. It was also shown that ETT plays a crucial role in electronic band structure via the association with the topology of the Fermi surface (FST)^29,30^. In effect, the relationship between ETT and FST provides new insight into the nature of the electronic structure of Li_5_MoH_11_, as will be discussed later. We also support results of electronic band structure and electron localized function by demonstrating the projected crystal orbital Hamilton population (pCOHP) method.

## Results and discussion

**Figure 1:**
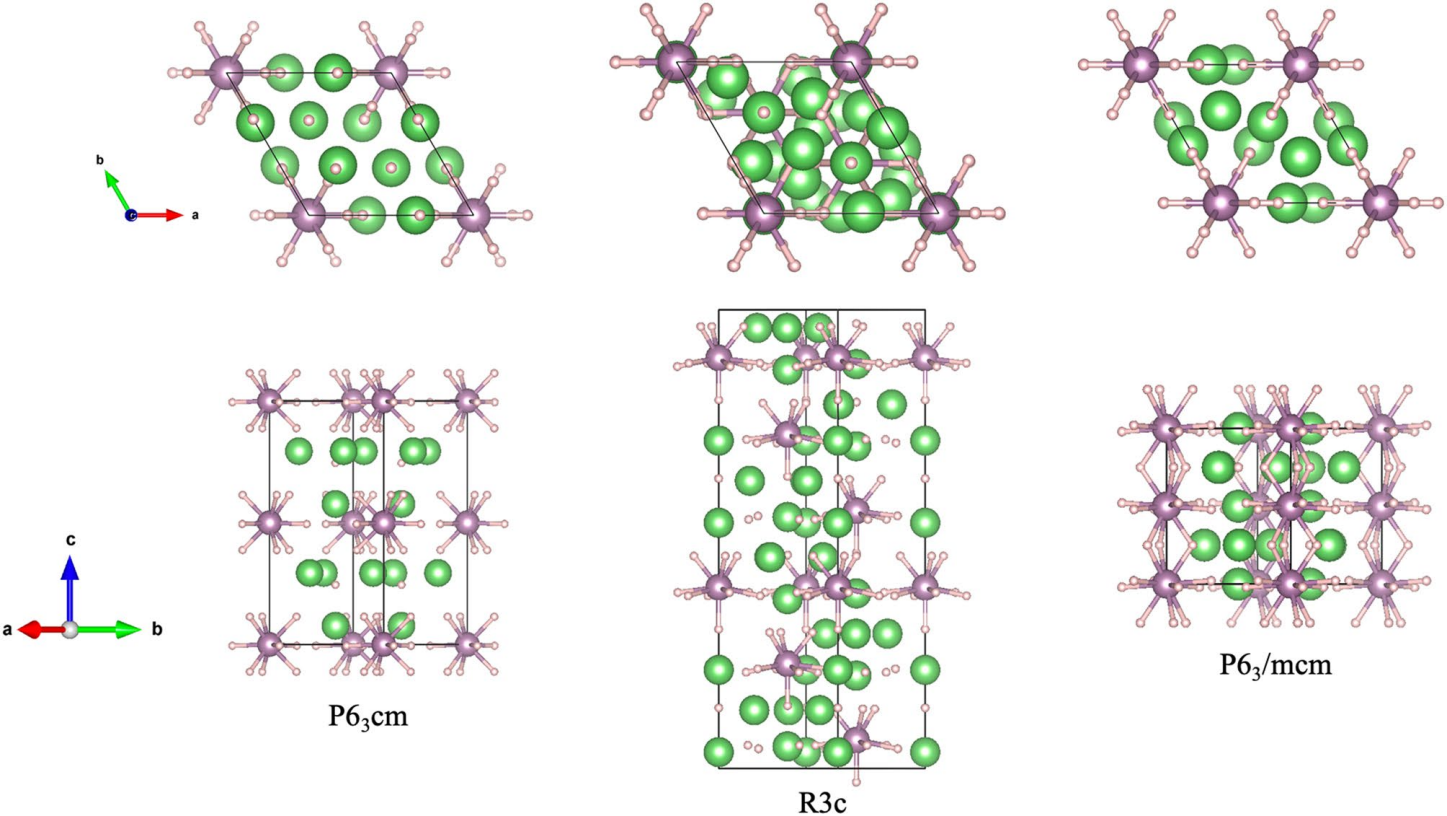
The crystal structures of Li_5_MoH_11_. The green, purple, and pink spheres represent, respectively, Li, Mo, and H atoms. (drawn by VESTA (ver. 3.4.7)^65^ (URL https://jp-minerals.org/vesta/en/download.html)).

We used USPEX to predict the crystal structure of the hydrogen-rich metal Li_5_MoH_11_ under high pressure and found that there exist two low-enthalpy structures adopting structurally identical hexagonal structures with *R*3*c* and P6_3_/mcm space groups. The predicted structures are shown in Fig. 1, while the optimized structural parameters for the *R*3*c* and the P6_3_/mcm structures are presented in Table 1. Subsequently the calculated structural parameters have been fitted to the Birch-Murnaghan equation of state (EOS), as summarized in Table 2. We initially conjectured that Mo^−^ might accept electrons from the surrounding Li^+^ cations under pressure^23^. Also, Mo atom is reported to have an unusual spin-coupling that eventually modifies the oxidation state^31^. We thus applied the GGA and GGA+$U_{\text {eff}}$ methods to investigate the possibility of this effect. In both cases, Mo^−^ was found to not accept any electron from the surrounding Li^+^ cations due to the fact that the predicted structure of Li_5_MoH_11_ structure is verified as being nonmagnetic (NM), since its magnetization is equal to zero. The selected value of $U_{\text {eff}}$ plays a decisive role in structural stability as well^32–35^. In the case of Li_5_MoH_11_, we thus evaluated the corresponding structural phase transition and electronic properties without incorporating $U_{\text {eff}}$.

**Table 1:** Structures of Li_5_MoH_11_.

Space group	Pressure (GPa)	Lattice parameters Å, $^{\circ }$)	Atomic coordinates (fractional)
R3c	60	a = 5.104 b = 5.104 c = 18.990	Li1 (0.989, 0.564, 0.960)
		$\alpha$ = 90 $\beta$ = 90 $\gamma$ =120	Li2 (0, 0, 0.714)
			Li3 (0.667, 0.333, 0.869)
			Mo1 (0, 0, 0.895)
			H1 (0.317, 0.987, 0.709)
			H2 (0.876, 0.540, 0.796)
			H3 (0.345, 0.002, 0.883)
			H4 (0, 0, 0.803)
			H5 (0.333, 0.667, 0.798)
P6_3_/mcm	160	a = 4.644 b = 4.644 c = 5.638	Li1 (0.667, 0.333, 1)
		$\alpha$ = 90 $\beta$ = 90 $\gamma$ =120	L2 (1, 0.587, 0.750)
			Mo1 (0, 0, 1)
			H1 (1, 0.221, 0.750)
			H2 (0.634, 0, 1.024)
			H3 (0.333, 0.667, 0.75)

**Table 2:** The structural parameters calculated by the Birch–Murnaghan equation of state.

Space group	V$_{0}$ (Å$^{3}$)	B$_{0}$ (GPa)	Method
P6_3_cm	151.1	21.9	Without ZPE
P6_3_cm	133.9	42.4	ZPE
R3c	144.3	21.8	Without ZPE
R3c	166.5	10.5	ZPE
P6_3_/mcm	109.7	51.5	Without ZPE
P6_3_/mcm	97.3	114.8	ZPE

**Figure 2:**
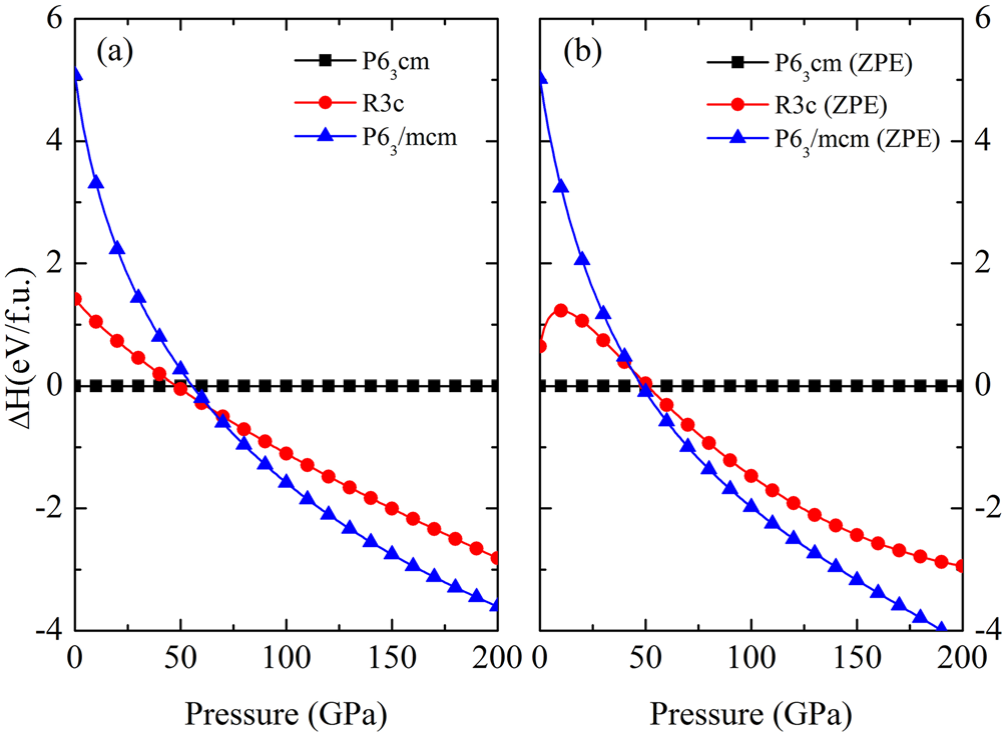
(a) The relative enthalpy as a function of pressure of Li_5_MoH_11_ without ZPE and (b) the relative enthalpy as a function of pressure of Li_5_MoH_11_ with ZPE.

**Figure 3:**
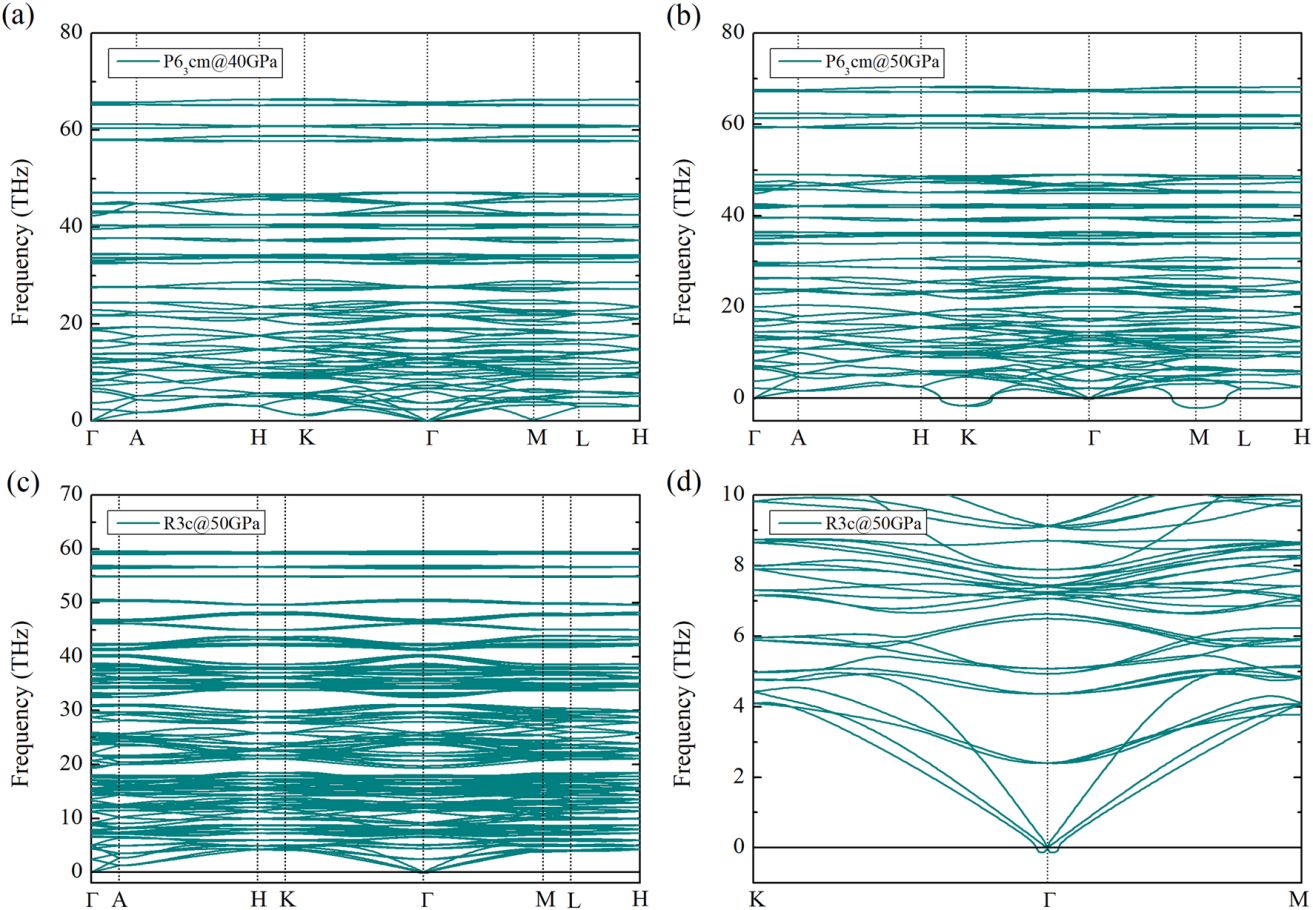
The phonon dispersion of Li_5_MoH_11_ (a) P6_3_/cm at 40 GPa. (b) P6_3_/cm at 50 GPa (c) *R*3*c* at 50 GPa (d) soft-mode at the $\Gamma$–point of *R*3*c* at 50 GPa.

The structural phase transitions of Li_5_MoH_11_ are presented by the relative enthalpy, which is obtained from the difference between the enthalpy calculated and that of the P6_3_cm structure, as a function of pressure. According to our calculations, the P6_3_cm structure transforms into the *R*3*c* structure at 48 GPa, followed by the existence of the P6_3_/mcm structure at 64 GPa, as shown in Fig. 2a. Addition to this, the corresponding structural stability is further confirmed by the incorporation of the zero-point energy (ZPE) of the nuclei estimation, indicating that the *R*3*c* structure is not energetically favored at all throughout the whole pressure range, as shown in Fig. 2b. Rather, the P6_3_cm crystal only transitions into the P6_3_/mcm structure at 48 GPa, which was previously pointed out that the *R*3*c* structure becomes unstable when taking into account the effect of ZPE^36–38^. The P6_3_/mcm structure, therefore, is thermodynamically stable over a wide range of pressures. When it comes to dynamical stability, the harmonic approximation incorporating ZPE of nuclei scheme is used to investigate the nature of the lattice dynamics of Li_5_MoH_11_. Reported in Fig. 3a, the phonon dispersions accounting for P6_3_cm structure at 40 GPa was computed and the structure is demonstrated to be stable, whereas upon a compression up to 50 GPa this symmetry is dynamically unstable due to the presence of negative frequency branches, as evidenced in Fig. 3b. In effect, the P6_3_cm structure has a tendency to transform into the P6_3_/mcm structure at this very pressure. The inclusion of ZPE calculation apparently plays a crucial role in determining the stable structure of Li_5_MoH_11_ under pressure, as previously demonstrated in other metal hydride systems^39,40^. Regarding the *R*3*c* phase, the responsible pressure-dependent phonon dispersion displays a tiny soft-mode at the $\Gamma$-point which is shown in Fig. 3c, accompanied by a zoomed-in $K-\Gamma -M$ path in Fig.3d. This suggests a possibility of becoming a meta-stable structure for the *R*3*c* phase, while the really stable phase belongs to the P6_3_/mcm structure.

**Figure 4:**
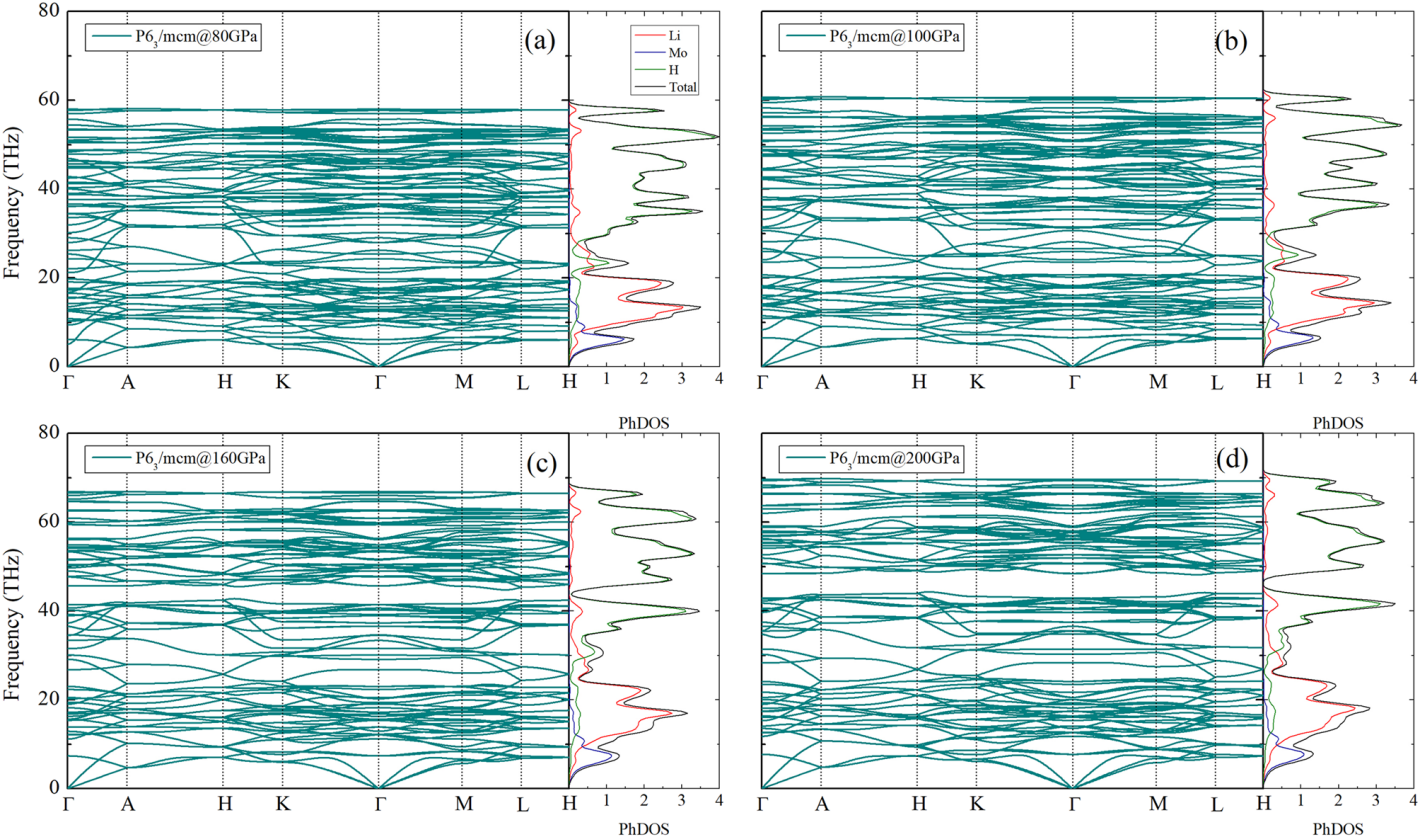
The phonon dispersion of Li_5_MoH_11_ (a) P6_3_/mcm at 80 GPa, (b) P6_3_/mcm at 100 GPa, (c) P6_3_/mcm at 160 GPa, and (d) P6_3_/mcm at 200 GPa, respectively.

**Figure 5:**
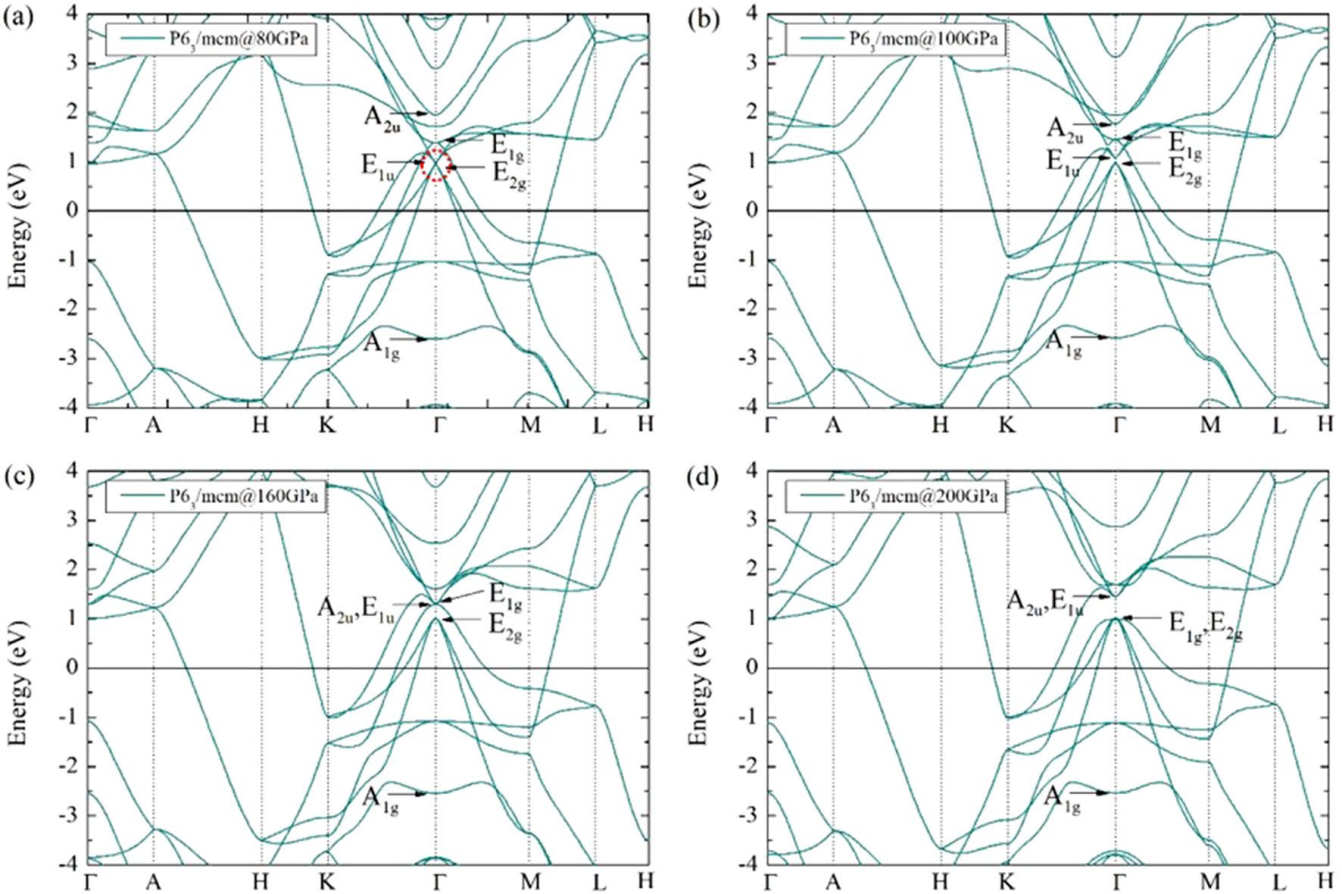
The band structure of Li_5_MoH_11_ (a) P6_3_/mcm at 80 GPa, (b) P6_3_/mcm at 100 GPa, (c) P6_3_/mcm at 160 GPa, and (d) P6_3_/mcm at 200 GPa, respectively.

Upon a series of compression, the P6_3_/mcm structure is clearly confirmed to be dynamically stable by the absence of negative frequency in the phonon spectra, as can be seen in Fig. 4, which implies that the compression promoted the global minimum enthalpy of the structure. At this point, we found that the P6_3_/mcm structure is dynamically favored over the P6_3_cm and *R*3*c* structures under a wide range of pressure, i.e., beginning at 80 GPa. According to the phonon density of states responsible for both Li and H atoms at around 25 THz (Fig. 4b–d), it is obvious that there exists coupling phonon branches, while they each tend to separate and eventually result in the intermediate optical phonon modes. It is worth noting that there remains the plausibility for Li_5_MoH_11_ to have a phase transition due to the fact that it was previously observed to superconduct with two different superconducting phases at 160 GPa^20^. At this point, however, it is difficult to verify whether there exists another phase of Li_5_MoH_11_ aside from the P6_3_/mcm structure, which, as a consequence, is expected to undergo the isostructural second-order phase transition above 160 GPa, as previously pointed out by means of the appearance of the intermediate optical phonon modes^41^ .

The electronic band structures of the P6_3_/mcm Li_5_MoH_11_ under varying pressures are plotted in Fig. 5a–d. The Mo atom’s 4d orbitals split into two energy levels, i.e, E$_{2g}$ (d$_{xy}$, d$_{x^{2}-y^{2}}$) and E$_{1g}$ (d$_{yz}$, d$_{xz}$), whereas the Li atom’s 2p orbitals yield the E$_{1u}$ (p$_{x}$, p$_{y}$) and the A$_{2u}$ (p${_z}$) orbitals as well as into the A$_{1g}$ orbital arising from the H atom’s 1s orbital. The band structure evidences a coupling between the E$_{2g}$ of Mo atom and the E$_{1u}$ orbitals of Li atom and reveals a Dirac-like cone, as also found recently^42,43^, at the $\Gamma$-point in Fig. 5a, which is originated from the coupling between the $\delta$- and $\pi$-bonds. Upon an increase of pressure, particularly at 100 GPa (see Fig. 5b), the separation of the E$_{2g}$ orbitals of Mo and the E$_{1u}$ orbitals of Li become apparent. This implies the electronic topological transition, which is referred to as the Lifshitz transition^44^. The following dispersion (see Fig. 5c) displays a downward shift of the A$_{2u}$ orbitals of Li, which in turn leads to the coupling between the E$_{1u}$ orbital of Li and the E$_{1g}$ orbital of Mo that exhibit the $\sigma$–$\pi$ bonding, at 160 GPa, when eventually the latter is roughly level with the E$_{2g}$ orbitals of Mo atom at 200 GPa also exhibiting $\sigma$–$\delta$ bonding. Likewise, the A$_{2u}$ orbitals of Li continue to couple with the E$_{1u}$ orbitals of Li despite the increase of pressure, which results in $\sigma$–$\pi$ bonding. As mentioned earlier, isostructural second-order phase transition induced by the P6_3_/mcm structure can be made possible by the Lifshitz transition. Moreover, the electronic band structures show no sign of flat bands near the Fermi level. Even though a set of flat bands can be spotted near the Fermi level as well as the presence of van Hove singularities (vHs) within H_3_S^13^, YH_6_ and YH_10_^14^ systems, leading to the possibility of achieving high values of $T_{c}$, this can be alternatively explained by the shape of the electronic band structure or density of states of our system. Our system’s electronic structures are similar to those found in YSH_6_^15^, LaSH_6_^15^, CeH_9_^16^, and CeH_10_^19^, in terms of steep branches near the Fermi level. However, we found that Li_5_MoH_11_ does not exhibit high-temperature superconductivity. As an explanation, the energy level of the H atom (the A$_{1g}$ orbitals) is lower than the Fermi level by -2.605 to -2.538 eV at 80 to 200 GPa, respectively, which further supports the implication that the high-pressure phase observed by experiment does not give high-temperature superconductivity^20^.

**Figure 6:**
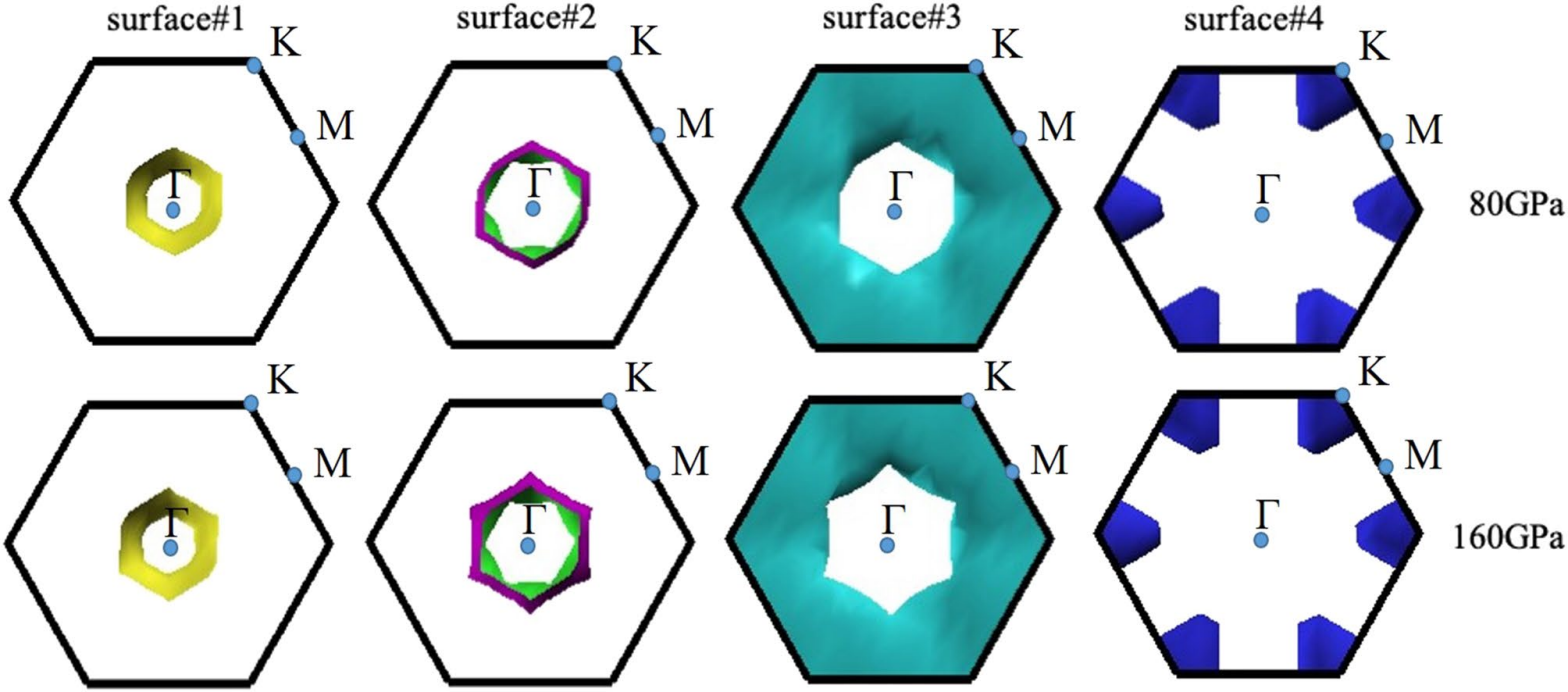
Sketch of FST of P6_3_/mcm at 80 and160 GPa, respectively (drawn by XCrySDen program (ver. 1.5.60)^66^ (URL http://www.xcrysden.org/Download.html#_toc_1)).

Another key finding that needs to be mentioned is the Fermi surface topology (FST). It is well-known that the FST is associated with an electronic topological transition (ETT), which originates from a given electronic band structure^24–28^, that plays a key role in promoting the second-order phase transition^45^. This was previously emphasized in previous studies^29,30^ the change of material’s topology is promoted by a compression. As depicted in Fig. 6, the characteristics of the FST clearly increase the likelihood of isostructural second-order phase transition: there are individual variations in surface#2 and surface#3 at 160 GPa , compared to those at 80 GPa. The topological changes in FST corresponds to the electronic band structures calculated. Thus the isostructural second-order phase transition of Li_5_MoH_11_ can be described by the presence of ETT. More interestingly, surface#2 and surface#3 both form the Fermi surface nesting at the pockets surrounding the $\Gamma$-point at 160 GPa, holding a vital clue to superconductivity^29^.

**Figure 7:**
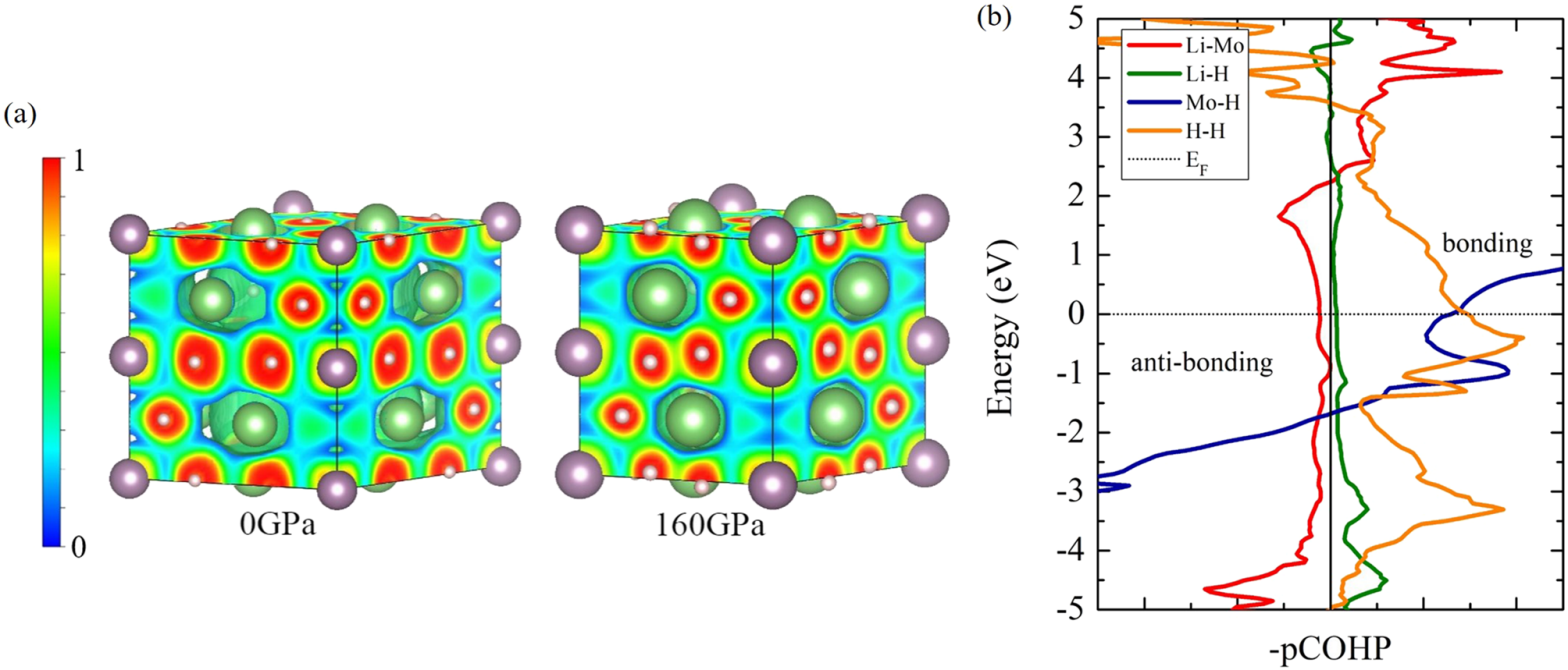
(a) The 3D-electron localization function (ELF) in Li_5_MoH_11_ structure at 0 and 160 GPa, respectively. (drawn by VESTA (ver. 3.4.7)^65^ (URL https://jp-minerals.org/vesta/en/download.html)) (b) Projected crystal orbital Hamilton populations (pCOHPs) in Li_5_MoH_11_ structure at 160 GPa.

The likelihood of finding an electron in the neighborhood space of Li_5_MoH_11_ can be measured by the electron localization function (ELF)^46^, as reported in Fig. 7a. The tendency of electron localization in the P6_3_/mcm structure is described by uniform electron gas of the same density^19,47–50^. The calculated ELF reveals a set of chemical bonding at 0 GPa. The distances between the first (Li–Mo), second (Li–H), third (Mo–H), and fourth (H–H) nearest neighbors (NN) read 2.94826 Å, 1.83412 Å, 1.90874 Å, and 2.24193 Å, respectively. There exists electronic probability weakly accumulating between the first NN Li–Mo, the second NN Li–H, and the third NN Mo–H, particularly the bonding between the fourth NN H–H which indicates that it is likely to be a strong bonding at 0 GPa. Upon further compression up to 160 GPa, the ELF displays that the distance between the first NN Li–Mo, second NN Li–H, third NN Mo–H, and fourth NN H–H are 2.38216 Å, 1.55862 Å, 1.70037 Å, and 1.27095 Å, respectively. It is worth noting that there is also a strong bonding between H and H, and the increase of electronic distribution between Mo and H at 0 GPa, implying that H–H bonding is likely to be a strong covalent bonding. We further investigated the nature of the chemical bonding near the Fermi level by with the aid of the projected crystal orbital Hamilton populations (pCOHP) calculation, which enables the determination of anti-bonding and bonding characteristics, e.g., covalent bonds, along energy range^51–53^. Illustrated in Fig. 7b, it is obvious that the projected wave function of the Li–Mo bonding displays anti-bonding, in a good agreement with ELF and corresponds to anti-bonding in the electronic band structure^54^. Our calculations also reveal that the E$_{1u}$ orbitals and the E$_{1g}$ orbitals exhibit the $\sigma$–$\pi$ bonding, by a determination of the electronic band structure. The projected wave function of the Li–H bonding is proved to be an ionic bond, which is confirmed by the ELF calculation, while those of the Mo–H and H–H bondings are indicated to be covalent bonds. In particular, the ELF calculation remarkably suggests a a strong covalent bond as a result of the H–H bonding. So, the characteristic of the Li–Mo anti-bonding implies that Li_5_MoH_11_ cannot achieve a high value of $T_{c}$.

## Conclusion

In this work, we identify the high-pressure phases of Li_5_MoH_11_ by performing an evolutionary searching. Our calculations show that by incorporating the zero-point energy evaluation the P6_3_/mcm structure is thermodynamically and dynamically favored over the P6_3_cm and *R*3*c* structures above 50 GPa The perspective of theoretical inspection points out that the P6_3_mcm structure exists under high pressure adopting the hexagonal basis. Phonon dispersion calculations and the electronic topological transition reveal that the P6_3_/mcm structure exhibits an isostructural second-order phase transition. Also, we have shown that the topology of the Fermi surface is associated with the electronic band structure, which nonetheless does not exhibit any flat bands near the Fermi level. This reflects that fact that Li_5_MoH_11_ does not have a high value of $T_{c}$. Here, our calculations support the experimental observations of $T_{c}$ in the previous study^20^. The nature of the chemical bonding is associated with the electronic band structure, implying that the characteristics of the chemical bonding entail the value of $T_{c}$.

## Methods

The searching for the structures of the hydrogen-rich metal Li_5_MoH_11_ was performed by USPEX^55^. In all subsequent generations, the random symmetric algorithm employed 40% heredity, 20% random symmetric, 20% soft mutation, and 20% transmutation operators in the pressure range from 160 to 200 GPa with structures containing up to four formula units. All structures were fully relaxed using the generalized gradient approximation of the Perdew–Burke–Ernzerhof (GGA-PBE) functional^56^ as the exchange-correlation functional. We selected the projector augmented wave (PAW) method^57^ to describe the core and valence electrons as well as the conjugate gradient scheme, as implemented in the Vienna ab initio simulation package (VASP)^58^. A plane-wave basis set up to a cutoff energy of 700 eV and a 10 × 10 × 4, 12 × 12 × 4, and 12 × 12 × 10 *k*-point meshes generated by the Monkhorst–Pack (MP) method^59^ were used for the P6_3_cm structure, the *R*3*c* structure, and the P6_3_/mcm structure, respectively. The pseudocore radii of Li, Mo, and H account for 1.7, 2.50, and 0.80 Bohrs, respectively, which are small enough to ensure that no overlap of spheres will occur under applied pressure. The zero-point energy of the nuclei (ZPE) is estimated within the harmonic approximation, which was included in the enthalpies as a function of the pressure. All values of enthalpy of all structures obtained were fitted by a Birch-Murnaghan equation of state (EOS). The dynamic stable structures were calculated by using the *ab initio* lattice dynamics with the supercell approach, as implemented in the VASP code together with the PHONOPY package^60^.The plane-wave energy cutoff of 60 Ry was selected as well as a 12 × 12 × 10 *k*-point mesh was used for the P6_3_/mcm structure. Based on the linear response theory^61^, the GGA+*U* was selected as a means to perform the Hubbard *U* calculation which is implemented in Quantum Espresso (QE) package^62^. The effective interaction parameters of which were tested to be $U_{\text {eff}}$ = $U-J$ = 1.2 eV. The projected crystal orbital Hamilton population^63^ (pCOHP) was used to describe the chemical bonding of the P6_3_/mcm structure, as implemented in LOBSTER code^64^.
